# Pregabalin-Induced Myopathy in a Double Lung Transplant Recipient

**DOI:** 10.7759/cureus.11935

**Published:** 2020-12-06

**Authors:** Vishwajit Hegde, Nidhi Shekar, Filip Garrett, Maher Baz, Michael Anstead

**Affiliations:** 1 Internal Medicine, University of Kentucky College of Medicine, Lexington, USA; 2 Pediatrics, Marshall University, Huntington, USA; 3 Pathology, University of Kentucky, Lexington, USA; 4 Cardiothoracic Surgery, University of Kentucky, Lexington, USA

**Keywords:** pregabalin, drug-induced myopathy, myositis, polypharmacy, necrotizing myopathy, lung transplant, cystic fibrosis

## Abstract

Pregabalin is a gamma-aminobutyric acid (GABA) derivative that was commercially approved by the Food and Drug Administration (FDA) in 2004. It is commonly used in the treatment of diabetic neuropathy, peripheral neuropathy, and spinal cord injury. We present the case of a 36-year-old Caucasian male double lung transplant recipient who presented with an 18-month history of fatigue and muscle weakness. He had elevated creatinine kinase level and his muscle biopsy showed evidence of drug-induced myopathy that improved after the cessation of pregabalin. We present a case of drug-induced myopathy as a rare complication of pregabalin therapy in a double lung transplant recipient.

## Introduction

Pregabalin is a gamma-aminobutyric acid (GABA) derivative that was first approved by the Food and Drug Administration (FDA) in 2004 in the treatment of neuropathic pain [1] and has since then emerged as a popular drug used in the management of diabetic neuropathy, post-herpetic neuralgia, and spinal cord injury and as adjunctive therapy in the treatment of fibromyalgia and partial-onset seizures. Pregabalin acts as a ligand on the a2 delta subunit on certain voltage-dependent calcium channels, inhibiting their action [2]. Pregabalin is dosed orally with up to 90% bioavailability. The medication is generally well tolerated. Although uncommon, the side effects that may occur include dizziness, somnolence, lower limb edema, mouth dryness, infection, and weight gain [3]. Myopathy associated with pregabalin use has rarely been reported. Previous reports of myopathy were associated with statin use [4,5], in the setting of sarcopenia or the setting of the recreational use of supra-therapeutic doses of pregabalin [6].

## Case presentation

A 36-year-old Caucasian male, with a past medical history of bilateral lung transplant for cystic fibrosis in 2017 complicated by a rejection episode, cystic fibrosis-related diabetes, hypertension, pancreatic insufficiency, anxiety, gastroesophageal reflux disease, chronic rhinosinusitis, and peripheral neuropathy presented with an 18-month history of progressive muscle weakness. The patient had a double lung transplant three years prior to the start of the muscle weakness. He had excellent exercise performance from a few months post-transplant, riding a road bike regularly and even participating in the Transplant Olympics. He experienced an episode of rejection about three years and three months after his initial transplant and required treatment with anti-thymocyte globulin. Although lung function was reduced after his rejection episode, he was still biking regularly up to 15 miles at a time. A few months after this episode of rejection, he developed some peripheral neuropathy symptoms, and therapy with pregabalin at a dose of 100 mg twice daily was initiated. Weakness started within two months after pregabalin therapy was initiated and slowly progressed. He was no longer able to ride a bike and had difficulty rising from a squatted position, or standing up from a chair. He denied shortness of breath, chest pain, peripheral edema, orthopnea, or paroxysmal dyspnea. There were no symptoms of hypothyroidism and thyroid function was normal. His daily medications included mycophenolate, tacrolimus, prednisone, pregabalin azithromycin, pramipexole, ursodiol, trimethoprim-sulfamethoxazole, metoprolol, omeprazole, oxycodone, ativan, melatonin, simethicone, calcium, sertraline, Humalog insulin, pancrelipase, magnesium oxide, methylcobalamin, a multivitamin tablet, and tramadol on an as-needed basis.

**Figure 1:**
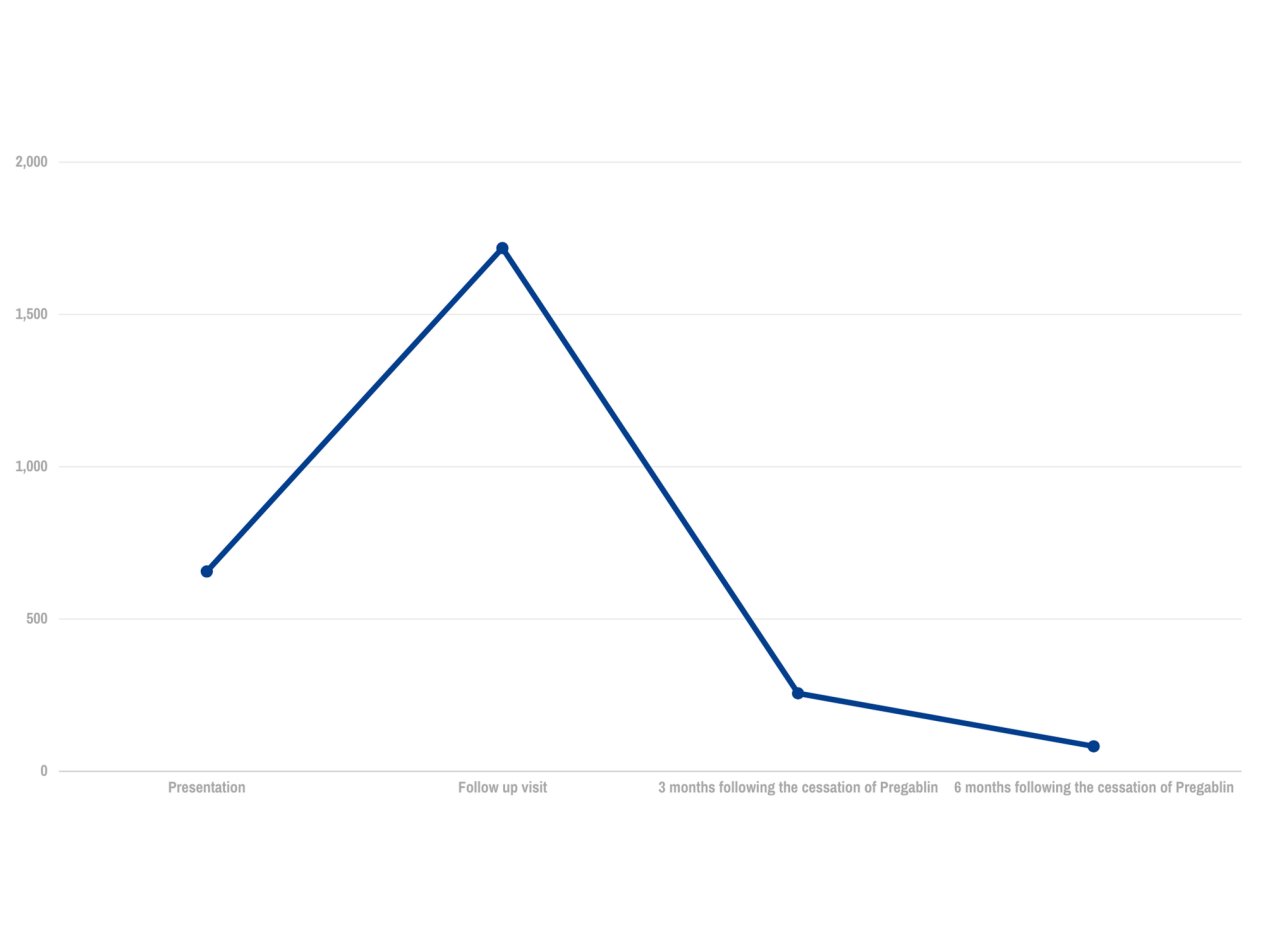
CPK level trends

Vital signs included a blood pressure of 120/82, a heart rate of 67, a respiratory rate of 18, and a temperature of 99 F. He weighed 76.6 kilograms with a BMI of 21.6, his oxygen saturation was 97% on room air. His general build and nourishment were normal, his fingernails showed mild clubbing. His neurological exam showed the presence of mild proximal muscle weakness; His heart had a regular rate and rhythm without murmurs. Peripheral pulses were normal. Lungs were clear to auscultation bilaterally. Laboratory testing revealed an elevated level of creatinine kinase and raised AST and ALT levels. The patient's pulmonary function tests showed a mild decrease in his FEV1 and FVC which subsequently improved while his muscle fatigue continued to worsen. A bronchoscopy performed to investigate his reduced exercise tolerance showed some purulent secretions and cultures grew methicillin-sensitive Staph aureus. Biopsy performed showed no evidence of rejection (ISHLT Grade A0, B0). Repeat labs drawn the next month showed a persistently elevated creatinine kinase level (Figure 1).

**Figure 2:**
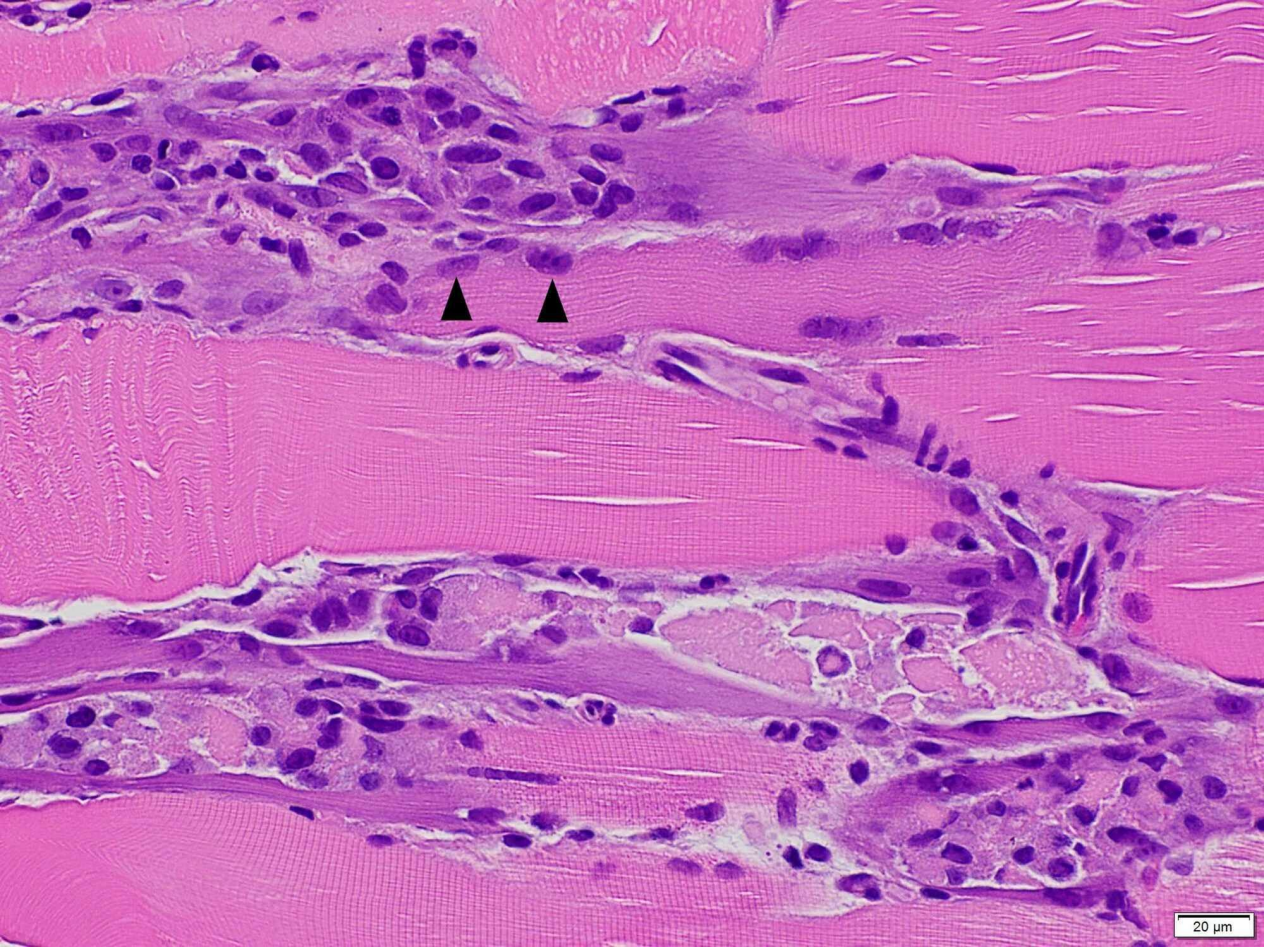
Left thigh skeletal muscle biopsy showing degenerative muscle fibers with macrophage infiltration (black arrows)

A muscle biopsy was subsequently performed for further assessment. It showed scattered degenerating and necrotic muscle fibers with an associated macrophage infiltrate and phagocytic response. A prominent acute neutrophilic or chronic lymphocytic inflammatory reaction was not seen and centrally vacuolated myofibers were not identified. Taken together with the clinical history, these histologic findings were consistent with a drug-induced acute necrotizing myopathy (Figure 2).

Upon reviewing the patient's medication for potential causes, mycophenolate and tacrolimus were viewed as the most likely causative agents, and the decision made to switch to cyclosporine and sirolimus. The patient did not report any improvement in his symptoms over a three-month time period. Creatinine kinase levels continued to rise despite the change in his medication regimen and the patient grew increasingly despondent. The decision was made to discontinue pregabalin. The patient noted a dramatic improvement in muscle weakness over the next few weeks and the creatinine kinase levels improved from 1023 U/L to 256 U/L (normal range: 49-320 U/L) within a month time period. Within 3 months, the patient was now able to walk on the treadmill for a half-hour and ride his bike again.

## Discussion

Therapeutic agents when used alone or in combination may inadvertently cause drug-induced muscle injury. Drug-induced myopathy should be suspected when a patient without preexisting muscle complaints presents with an acute or subacute onset of symptoms such as muscle weakness, myalgia, muscle tenderness, creatinine kinase elevation, or myoglobinuria occurring after the administration of a drug. The clinical spectrum of drug-induced myopathy can range from mild muscle weakness and inconvenience to permanent muscle damage and disability. Drug-induced muscle damage can be clinically classified into painful, as typically seen with statins, zidovudine, cyclosporine, clofibrate, and painless, as seen in myopathy associated with colchicine, corticosteroids, chloroquine derivatives, beta-blockers, and certain antibiotics [7].

Drugs can cause myopathy by (a) directly affecting muscle organelles such as the lysosome, mitochondria, and the cytoskeleton, (b) altering the electrolyte levels, nutritional supply, temperature causing secondary muscle injury, and (c) altering muscle antigens, thereby causing immunologically mediated injury [8].

Bilateral lung transplants are an accepted treatment for patients with cystic fibrosis with advanced lung disease. Lung transplantation provides a significant quality of life improvement for these patients. The long-term survival of patients with cystic fibrosis who undergo transplantation is excellent with a median survival rate of 10 years at experienced centers [9]. Post-transplant, patients are placed on a complex drug regimen to prevent rejection and opportunistic infection may of which can be potentially myotoxic. Many patients may be on medications for other comorbidities and diagnosing and identifying the agent responsible for drug-induced myopathy may prove challenging.

In cases where the diagnosis of drug-induced myopathy is uncertain or when the differential diagnosis is very wide, obtaining a muscle biopsy can provide definite evidence of muscle injury and it potentially provides clues to the etiological agent [10]. Our patient was on mycophenolate and tacrolimus which could potentially cause myopathy [11,12]; however, the patient's symptoms did not resolve when the patient was switched to cyclosporine and sirolimus. Given that (a) the patient did not have any muscle weakness prior to the initiation of pregabalin, (b) improved rapidly following the discontinuation of the drug, (c) the patient did not develop myopathy again when he was switched back to his tacrolimus afterward for different reasons, pregabalin was the cause of the myopathy in this patient. We can find only four reported cases of pregabalin induced myopathy in the literature, three cases were in the setting of concomitant statin use and one in a psychiatric patient that had consumed very high doses. The Naranjo causality algorithm indicates a probable relationship [13] between pregabalin and the development of myopathy in this case (Naranjo score - six).

## Conclusions

Pregabalin is a widely used drug in the treatment of neuropathic pain. As patients that are transplanted become more medically complex, it is important to recognize the potential drug interactions and adverse drug effects. It is important to recognize pregabalin as a potential cause of myopathy. Patients on pregabalin that develop muscle symptoms should be evaluated for potential myopathy and discontinuation of the drug should be considered.
